# A qualitative analysis of the enforcement of the regulation of nutrition and health claims made for foods and its implications for health

**DOI:** 10.1371/journal.pone.0201178

**Published:** 2018-07-24

**Authors:** Ajay Patel, Sumaiya Patel, Rebecca Gregg, Laura O’Connor

**Affiliations:** Department of Health Professions, Faculty of Health, Psychology and Social Care, Manchester Metropolitan University, Cavendish Building, Manchester; Sefako Makgatho Health Sciences University, SOUTH AFRICA

## Abstract

In common with local government organisations across the world, local authorities in the UK have responsibility for promoting health. A key part of this function is the frontline enforcement activities of officers responsible for compliance with health and nutrition claims. This study identifies attitudes, values and practices of enforcers: namely trading standards and environmental health officers, when faced with the problem of non-compliance with the Regulation. Semi-structured interviews with frontline enforcers from local authority regulatory services to investigate challenges with the enforcement of Regulation (EC) 1924/2006. Twenty participants were interviewed; sixteen were based in North West England and two in the North and two in the South of England. The participants were selected for their specialist knowledge and experience of enforcement of nutrition and health claims. Regulation (EC) No. 1924/2006 on nutrition and health claims presents particular challenges for enforcers seeking to apply an optimal strategy to flawed regulatory design. As with other regulations, when faced with non-compliance, enforcers, specifically trading standards and environmental health officers have a wide discretion to determine their response: ranging from the deterrent or accommodative styles of enforcement. The participants reported using advice rather than action and by doing so confronting their bifurcating identity of prosecutor and advisor. Enforcers used advice as a regulatory tool in enforcing the law relating to nutrition and health claims.

## Introduction

Along with other market driven consumer protection in the EU and internationally[1], the aim of the Regulation (EC) No. 1924/2006 on nutrition and health claims [2] (the Regulation) is to ensure that consumers are provided with adequate and reliable information be able to make informed food choices [3]. The Regulation defines a ‘nutrition claim’ as a claim that a food has particular beneficial nutrition properties such as ‘high in fibre’. A ‘health claim’ is a claim that health benefits can result from consuming a food, such as ‘helps build strong bones’.

An ‘information asymmetry’ as defined by Stigler [4] exists between suppliers and consumers of food that justifies regulatory intervention[5]. This is because consumers are not well placed to judge the nutritional and health characteristics of a food as such attributes are latent and undiscoverable[6]. Therefore issues arise where more readily assessed attributes such as price and sensory qualities of a food dominate consumer decision-making [7], the outcome of which may lead to poorer nutritional, health and obesity status for consumers. Therefore, the misinformed decisions of consumers may jeopardise their health and welfare. Where a nutrition or health claim is made, the Regulation requires that such a claim ‘shall be based on and substantiated by generally accepted scientific data’ (Article 6).

The rationale of the Regulation [2] is to provide information in order to correct the quality signalling problem rather than to impose a restriction, limit or ban on the sale of nutritionally poor foods [8]. The efficacy of the Regulation may depend on how enforcers go about the operational task of enforcement. Enforcement might be examined through the economic perspective. From this standpoint, the benefit of food law enforcement is an economic good like any other.

The purpose of the study was to identify attitudes, values and practices of enforcers: namely trading standards and environmental health officers, when faced with the problem of non-compliance with the Regulation. There were similarities between the enforcement of the Regulation and the broad range of other legislation enforced by officers.

## Methodology

The study was a phenomenological investigation into the experiences and beliefs of enforcers, where the themes were confined by a reductive approach. A non-probability purposive sample [9] was chosen to capture those who were most likely to provide useful insights to the practice of enforcement in the relevant area. Specifically, a number of the trading standards officers among the sample population were identified by their trade body, the Trading Standards Institute and by each other as experts in health and nutrition claims rather than in many of the other areas of enforcement in which environmental health and trading standards officers are commonly involved. The environmental health officers were identified as having similar expertise by referral. The participants were approached by an invitation sent by email. Of the 25 participants contacted, 20 agreed to take part. All 20 were interviewed notwithstanding that data saturation was reached before completion, that is, where the a priori themes were adequately represented in the data.

Ethical approval was obtained by Manchester Metropolitan University prior to data collection. In this respect, the key concerns for participants were preserving the integrity of their investigations and commercial sensitivity of the businesses who they were investigating. Assurances of confidentiality were provided and the data were anonymised at transcription to prevent identification of participants or food suppliers. The participants provided their consent to be interviewed by email.

In the course of this study, eighteen semi-structured interviews were conducted with twenty respondents. Pseudonyms have been adopted to refer to the participants to conceal their identities (see Table 1).

**Table 1:** Description of interviewees and their specialist areas of regulation.

Name	Area of regulation	Officer, team leader or management responsibility
Chloe	Trading Standards	Supervisor
Millie	Trading Standards	Manager
William	Trading Standards	Supervisor
Ethan	Trading Standards	Officer
Jessica	Environmental Health	Supervisor
Julie	Trading Standards	Officer
Sophie	Environmental Health	Manager
Scarlett	Trading Standards	Officer
James	Trading Standards	Officer
Charlotte	Environmental Health	Officer
Jacob	Environmental Health	Officer
Dexter and Theo	Environmental Health, Trading Standards	Director, Manager
Isaac	Trading Standards	Officer
Mia and Cate	Trading Standards, Trading Standards	Officer, Supervisor
Ruby	Trading Standards	Supervisor
Sienna	Trading Standards	Officer
Samuel	Environmental Health	Manager
Amelia	Trading Standards	Supervisor

The interviews were conducted in meeting rooms at the offices of the respondent’s employer: the regulatory services department of a local authority. The interviews were of a duration of between 45 and 90 minutes. The interviews were recorded on a digital recording device after obtaining the respondent’s consent.

All of the above officers were employed in authorities in the North West of England except for Dexter and Theo who are based in the North and William and Julie who were attached to authorities in the South. Each of the interviews were conducted, transcribed and coded by one investigator, AP. The transcripts were uploaded into NVivo (version 10, QSR International) and a thorough process of data coding and identification of themes completed. The coding process involved identification of a significant statement and encoding it [10] and a useful code was one that captured the qualitative richness of the phenomenon. The themes were derived at deductively with reference to the themes in the interview guide. Themes and the relationship between these themes were systematically organised into thematic networks summarising the main influences on enforcers [11].

## Results

While the general direction of the work was influenced by the literature and the broader themes might be said to exist a priori, a number of more detailed and particular themes emerged as a result of the analysis of the interview data. There was broad agreement among the interviewees in the major areas of contention and this lead to the emergence of themes. Some of the themes are explored in detail and form the basis of the discussion here and this is based on their relevance to the research question of how enforcers enforce the Regulation and their use of discretionary powers in taking enforcement action.

A notable theme which emerged from the data is the enforcement officers’ identification of themselves as prosecutors with sole and direct responsibility to the public: a view consistent with the deterrence model presented by Reiss [12] and developed by Hawkins and Hutter [13]. A further related theme was a view of enforcers as advisors to business. Such an approach is rooted firmly in the accommodative model of Braithwaite et al where officers seek to ‘educate, persuade and cajole’ [14]. In this capacity, the enforcer adopted techniques such as education, persuasion and negotiation to deal with cases. In this role, enforcers’ tactics were more informal.

Ethan captures the duality of his position as an enforcer and how he reconciles the functions:

We always have to play two roles. Supporting businesses and the enforcement but they do tend to blend together quite well I find. And most businesses want to be compliant, and they know that, so if you can help them be compliant then they’ll you know, go with you.

Traditionally enforcers have had an arm’s length relationship with business. However, Ethan, like his contemporaries, is not uncomfortable with modern enforcement practice where enforcers play a role in supporting businesses in their efforts to ensure compliance with the law. This would support the notion that ‘an advisory approach to regulatory enforcement may be a necessary component of an effective enforcement strategy’ [15].

Similarly, at the start of the interview where the enforcement officer is invited to describe their role and main duties, Chloe describes her view of her role primarily in terms of enforcement as her main purpose:

Well, enforcement is our main, we are regulators, enforcement is our main business advice and primary authority advice is probably secondary to that.

It is notable how Chloe distinguishes between the roles of enforcer and advisor. She sees herself as an advisor but able to switch from that to regulator. This is at odds with the theory that advice is an *integral* part of the compliance strategy as described by Law [15] but compatible with the regulatory pyramid of Ayres and Braithwaite [16–18] where enforcers move up and down the range of possible regulatory measures.

Later, Chloe describes how consumers might threaten to report matters to the local press:

Consumers would threaten companies by saying that they would report cases to us or that they would call the xxx News [the local newspaper]. Environmental health stories would often be front-page news. And if we didn’t take the action they wanted, they would report us to the paper

In her view, there is a public perception that she should be taking a deterrence-based approach as described by Reiss. It is not clear why Chloe feels that public expectations would require that she does not provide advice to business. This may be due to a failure by the public to appreciate the nature of the enforcer’s role and in turn, this may be attributed to the lack of communication by enforcers about their duties. Certainly, Chloe’s remark regarding consumers indicates that she faces some resistance to the idea of helping business and that she ought to police and prosecute non-compliant business. However, in her practice Chloe actually adopts an advisory role. In fact Chloe goes much further when she actually speaks of promoting economic growth as an objective in itself and part of her work rather than as a means of securing compliance: ‘*we’d give assistance with small businesses because we’ve got obviously priorities to promote prosperity and business growth in xxxx*.*’*

The role of economic regulators to deliver a competitive marketplace in order to provide an environment for business growth is well established [19,20]. The literature on enforcement practice does not however document the role played by a consumer protection enforcement agency in delivering business growth. To a degree, this may arise from the unique nature of local authority work where the authority has a duty to promote the welfare of its local residents including job creation.

Charlotte revealed that the provision of advice can provide the initial engagement between enforcer and a firm on the subject of the application of nutrition and health claims. When asked what experience, if any, she had of nutrition and health claims, she responded:

Some of the food manufacturers based in this area or other food businesses will ring up saying I want to make a claim on my label and I’ll give advice.

Amelia went on to provide specific instances of the cases of such advice:

We have provided advice to a sports supplements importer…We have also been involved in giving advice to an internet trader for vitamins.

The reference here is to advice provided in response to a request for assistance from a firm. In their paper *Really Responsive Regulation* Baldwin and Black stress the case for regulators to be responsive to not only the attitude of the firm but also the ‘operating and cognitive frameworks of firms’ [21]. In responding to the specific request for advice, enforcers or regulators avoid the ‘expensive process of shooting in the dark’ [21] as the advice is targeted to meet an expressed need.

In both deterrence and accommodative approaches, the enforcer sought to achieve the same goal: compliance with the regulation and therefore to stop the offending behaviour in the case in hand and to deter such behaviour in other cases. The roles of enforcer and advisor were not mutually exclusive; in fact, it was common for the enforcer to move from the position of advisor to prosecutor in the same case as lower level action failed and the case escalated to stronger measures. ‘*Initially advise them*, *yes*. *I mean if they don’t listen to advice then you can take more formal sanctions but most of them will comply’* Ethan. This confirms the notion of escalation up the regulatory pyramid of Ayres and Braithwaite et al [16].

### The enforcer as advisor–the nature of the relationship and its defining characteristics

Mia alludes to an established relationship between the enforcer as advisor and the business as client: ‘*Because we do have a number of manufacturers that we do advise generally on labelling*.*’*

Harrison et al found that the educative function ‘is increasingly focused upon the lower tier of food retail establishments, such as a butcher’s shop or a bakery’ [22]. There are now very few independent butchers and bakers on high streets. In the light of the change in this composition of the high street the current independent food businesses which are most prevalent are fast food takeaways and it was apparent from the data that these are now the focus of educative efforts of officers.

Charlotte distinguishes between the circumstances in which the Regulations will apply and where they will not. Using the term healthy as a general claim is not covered by the Regulations unless there is a specific claim which accompanies it. In her account, Charlotte also demonstrates an appreciation of the commercial imperative applied by the organisational structures within a business.

Dexter describes the challenges of providing practical advice based on complex legislation, which might be translated into action by the recipient:

I think understanding the accuracy of the health claims is complex. It’s not as easy as just looking and seeing oh that label needs to have a best before date or the use by date is wrong, it’s looking at the whole thing, and (a) it’s a health claim and (b) I need to go and check that to see if it’s correct so I need to go back onto the website so it’s not immediate. You need to go back and double check, the local website is (a) it’s a valid health claim and (b) it’s authorised and then go back to the business and say well we need to make some changes.

The provision of advice, albeit without charge, would potentially give rise to liability to the local authority for which it may be prudent to obtain professional indemnity insurance or ensure that it is protected under the terms of its existing cover. Such liability would be subject to the ‘Caparo’ test for negligence resulting in economic loss, that is the harm is reasonably foreseeable, a relationship of reasonable proximity exists between the parties and that it is just and fair to impose liability [23]. This raises the question of whether this might have the effect of formalising the relationship between enforcers and the regulated firm and inhibit the provision of advice. It would certainly put to the test the circumstances in which liability might arise.

For their part, officers are protected from liability under section 44 of the Food Safety Act 1990 where they act in good faith and in the course of their duties. The local authority would be vicariously liable and in the event that an action that an action is pursued against the officer for acting outside the course of their employment, the local authority may indemnify them.

The issue is most acute where the regulated firm is an SME which does not have the knowledge or awareness of the laws which apply to it and therefore are unlikely to have in place the resources such as financial commitment and management systems in place to ensure compliance. They are also unlikely to be able to afford hire such expertise. SMEs do not have the personnel or time or availability to be able to monitor compliance and to interpret law [24]. In such cases, the firm is reliant on the free advice provided by the local authority. If such a service is stopped or restricted to avoid liability for negligent advice than this may lead to more non-conformity in a sector where there is already significant breach.

We can see from below that Jessica appreciates the importance of consistent advice for businesses, particularly where they operate across local authority boundaries. Yapp and Fairman confirm this by their finding that where there was a follow up visit to the same premises and there was an inconsistency between the advice provided on each occasion that this would lead to a lack of trust:

EHPs [Environmental Health Professionals] were seen to act inconsistently, both within the individual business and between businesses. SMEs complained that different food safety requirements were made each time the premises was inspected, despite conditions remaining the same and the same EHP visiting. SMEs also believed that EHPs would forget or fail to enforce requirements made previously and therefore failed to take action…

Yapp and Fairman were concerned with inconsistency between repeat visits to the same business[25]. A similar concern was also raised by Cranston in 1979 at the general level of the exercise of unconfined discretion in *Regulating Business*: *Law and Consumer Agencies* [26]. The risk of inconsistency between different officers operating in different local authorities is significantly greater.

The primary authority process was established to ensure that the local authority advice across boundaries is consistent [27]. A recurring theme of the data was how businesses feel an acute sense of unfairness where it appeared that a competitor was able to make claim which they are advised not to, *‘They will say*, *x*, *y and z are doing this and we want to do it as well*. *How come they can do it*, *and we can’t*?*’* Jessica.

One of the methods used by local authorities to ensure consistency is the setting up of a primary authority relationship which provides notice to other authorities that advice has already been given. Interestingly, here Chloe also speaks of being a burden on business; not a position that one might ordinarily associate with an enforcement official:

There’s a section just for regulators on the BRDO [Better Regulation Delivery Office] website so I can go on that register and look for like a national supermarket and see if they’ve been advised on health claims. If I was to go and visit that supermarket, I have to look at the primary authority register to see if they’ve got a primary authority and I have to have regard to advice that’s already been given. **We do burden a business**, so like at xxx (major supermarket), if they’ve got 422 local authorities all going in, taking up the time of store manager, their primary authority could say, ‘no need to look at this area, we’ve audited it and we’re happy with it. All the primary authority partnerships across the country are listed on this website and regulators have a special log in to check what advice has been given. So if I was to do, say a xxx [name of major supermarket] inspection, I’d have to go and look at their inspection plan, any advice that had been given, to know where to direct my resources when I go into my local store.

Chloe seems concerned that the intricate nature of the regulation is not optimal rather than that there is any at all. The research evidence points to inefficiencies in command and control systems of regulation and a preference for market based regulation. Therefore, the questions are around when and how to regulate and how to avoid problems of regulatory capture [28]. Chloe’s suggestion is aimed at avoiding the inefficiencies of the regulation of individual outposts of a business with multiple branches.

The primary authority relationship where business receives advice in return for payment, may provide a level of indemnity which commercial advisors cannot provide, as Chloe explains:

And as much as we wanted to be able to say, yes, we couldn’t so we’ve not heard from them since. And the primary authority has gone, because that was, to be fair, I did say to them, if we can’t approve and give primary authority advice to say that you can use that, there’s very little point in us actually being the primary authority.

Where enforcers can provide an indemnity against action from either their own authority or from another authority, this provides a valuable benefit and assurance to business. This goes beyond the benefit that might be provided by an independent professional advisor such as a lawyer while raising the issue of liability for advice provided and the provision of a benefit which cannot be matched by any other provider. It also raises questions of the conflict of interest between the provision of paid for advice and assistance on the one hand and the duty to enforce against that same party (who turns from client to defendant), on the other.

Enforcers can demonstrate a surprising level of involvement with the commercial aims of the business as Scarlett shows:

I’m trying for them not to change their trademark, I know they want to keep their trademark. How can we go round–not circumvent the legislation obviously, but get to a point where they can continue to use it

There are specific provisions in the Regulation which make it clear that a claim contained within a trademark is subject to the same control as if it were made without the trademark. In other words, that the registration of a trademark does not provide immunity to a claim made within it. Local authority enforcers do have experience and knowledge of law for business. However, it would be unusual for that to extend to providing advice relating to intellectual property issues arising from the protection of trademarks and to provide advice about how the trademark might be protected.

### The limits of the advisory function

At times, there was an expectation from a business that enforcers would be on hand to provide advice whenever it was required. James describes that expectation:

certain companies would be phoning you all the time so you felt like you were doing their work for them which is ok but when we are stretched it’s not possible to do that as much so it’s something that’s in formation at the moment with primary authority, so we’ll see how it works out.

Enforcers would need to adopt strategies to manage the expectations of businesses because they were unable to deliver the service levels which business would have liked and possibly had become accustomed to from other providers. The problem of managing business expectations was most acutely felt following the severe cuts to local authority budgets imposed from 2009 onwards. James described the circumstances:

We’re short staffed now but we used to have a chap who was office based and people would send their labels for approval and raise issues they have and he could spend quite a lot of time doing it. We don’t provide that service any more.

It will be instructive to see whether the withdrawal of a label copy clearance service will lead to more ex post liability breach and with enforcers playing a lesser role in the provision of ex ante advice. The effect of withdrawing ‘free’ advice may implement a cost structure and more efficient distribution of resources and avoid the waste associated with services provided free at the point of use[29]. Alternatively, it may result in greater non-compliance and problems of consumer detriment that might have been avoided more efficiently by providing advice. This was raised as a concern by more than one of the interviewees.

### Advice as a preliminary step towards action

It was clear from all of the data that advice represents the first step in the ‘enforcement ladder’ with further steps becoming more formalised as Ethan says, *‘Initially advise them*, *yes*. *I mean if they don’t listen to advice then you can take more formal sanctions but most of them will comply*.*’*

In the majority of cases, matters will not proceed any further than the advice, ‘*most businesses are reasonable*. *We advise them ‘ you must get rid of this’ and they do or are in the process of doing so*.*’*

Ethan explains the broader position:

All enforcement tends to be a hierarchy of actions, we decide the proportion as best we can. And it ranges from just, you know, simple verbal advice up to a full prosecution. It sort of increases in more formality if you will, as you need to, if somebody doesn’t listen to written advice you give them, sorry verbal advice, then you tend to give written advice, then formal written advice…prosecution is the final resort.

Attempts to capture and illuminate the negotiation between enforcers and regulated firms have found this hierarchy of actions [14,30,31]. The theory of the ‘regulatory pyramid’ of a hierarchy of actions from negotiation to prosecution provides strategic level guidance on how enforcers should carry out their duties. This is consistent with the way in which enforcement is implemented as described by Ethan. Such a responsive approach to regulation is commonplace and involves regulators who enforce ‘in the first instance by compliance strategies, such as persuasion and education [but] apply more punitive deterrent responses (escalating up a pyramid of such responses) when the regulated firm fails to behave as desired’ [21].

In the pyramid of responses, as one regulatory intervention fails, the regulator moves upward to the next more serious level and as the risk subsides, the regulator should move back down to a lower level. In this way, the pyramid provides an inherently proportionate and reasonable exercise of power the justification of which is based on the failure of the less serious previous action.

The commercial response to the advice demonstrates the level of reliance placed on the officer’s judgement as shown by Charlotte:

The first step would be advisory. Then you would ask them to change the label. And most of the time they do. Sometimes they stop making the product altogether.

It is unclear whether the advice to change a label resulting in the withdrawal of the claim, and finally to stop making the product altogether, is wholly dependent on the opinion of the enforcement officer or whether the opinions of others have been sought. It is clear however, that business can place great reliance on the opinion of the officer and it plays a significant role in influencing the decision.

## Discussion

In relation to the enforcement of the Regulation, Hawkins and Hutter capture the central difficulty thus; ‘the task of a regulatory bureaucracy is, by various means, to induce a potentially unwilling business organisation to bear costs which it would in many circumstances not wish to assume’ [13]. The costs of compliance will not be incurred in the absence of coercion by enforcement officers. Such officers play a significant role in influencing the business to incur compliance costs and to do so in a way which provides the optimal level of protection for consumers.

While the emphasis of this study was on the enforcement of the Regulation, the study found evidence for both the deterrence and accommodative approaches of enforcement across the range of legislation. When interviewed, enforcers identified themselves primarily as enforcers whose first duty was to the public. A further related theme, which is not found in the literature, but which has more recently emerged is the view of enforcers as advisors to business. Such an approach is rooted firmly in the accommodative model of Braithwaite et al where officers seek to ‘educate, persuade and cajole’ [14]. In this capacity, the enforcer adopted techniques such as education, persuasion and negotiation to deal with cases. In this role, enforcers’ tactics were more informal. Such a step may be seen a further step along the accommodative path where enforcement officers are less enforcers in the traditional sense and they begin to take on the characteristics of advisors and advisors.

Marc Law raises the notion that ‘an advisory approach to regulatory enforcement may be a necessary component of an effective enforcement strategy’ [15]. The data in this study showed evidence of the engagement in advice as part of enforcement. One might have expected there to be some angst about fulfilling what appear to be conflicting roles, however respondents appeared to be able to switch between the two without any apparent difficulty.

In examining this advisory function, the communication between the firm and the regulator is found to demonstrate an awareness of the responses required to not only the needs and attitude of the firm but also the ‘operating and cognitive frameworks of firms’ [21]. Examples of this are evident in the commercial nature of the advice on claims for sports supplements made by internet-based businesses.

The data from the sample interviews of enforcers in this study shows how the roles of advisor and enforcer are taken on and adapted to suit the circumstances. This supports the notion of escalation up the regulatory pyramid of Ayres and Braithwaite et al [16]. Sometimes, even in the same case, enforcers would move up but less easily down the pyramid of regulatory responses.

The study found the relationship of local authority advisor and firm differed from that of the retained advisor and firm in some important respects. There was a willingness to challenge the advisor based on observations of what appeared to be inconsistent practice and the tolerance of non-compliant claims in circulation. This raises questions of fairness to which there appears to be no wholly satisfactory answer from enforcers. The conflict between the role of advisor and prosecutor is felt most keenly when faced with the prospect of action from another authority which challenges or undermines the advice provided to the firm. The overriding concern from firms however is for consistency and certainty for the advice which they receive. This places great pressure on enforcers to provide independent, bespoke, complex and commercially aware advice that will face up to scrutiny and possible challenge by experienced advisors. Not surprisingly, the data showed some faltering of confidence among officers (see Dexter above) particularly when faced with the absence of the coordination previously provided by Local Authorities Coordinators of Regulatory Services (LACORS).

The data were collected soon after the implementation of the Health and Social Care Act 2012 which imposes significant obligations on local authorities for improving the health of their populations. The responsibility lies primarily with a specialist public health team led by the director of public health supported by existing expertise in areas such as environmental health and trading standards. As such the study highlights the scope for a joined up and strategic approach within local authorities. Many of the officers interviewed reported being subjected to increasing demands from business for advice with expectations of service levels resembling those within commercial practice. Against a background of cuts in local authority services, this represents a further strain on resources. The difficulties to which it gives rise are underscored by the fact that, in the absence of a primary authority agreement, such advice is provided without charge.

## Conclusion

The study sought to identify attitudes, values and practices of enforcers: namely trading standards and environmental health officers, when faced with the problem of non-compliance with the Regulation. The interviews with enforcers revealed several themes; including that of the application of bureaucratic discretion, deterrence, accommodation and the enforcer as advisor, which were considered to be most relevant in influencing the type of action to take in applying their discretionary powers. The recommendations from this work would suggest the need to promote an awareness of the costs of the very broad nature of the work of enforcers and the extent to which their duties extend to an advisory function. Such insights can contribute to the understanding of the enforcement action for enforcers and health professionals and may be applied to other jurisdictions where enforcers enjoy similar discretion. A fertile direction for future research would be a comparative study to explore how enforcers in other jurisdictions approach similar problems.

## Supporting information

S1 Checklist COREQ checklist. (DOCX)
