# Supplementary material for: A qualitative analysis of the enforcement of the regulation of nutrition and health claims made for foods and its implications for health
Source: PLoS One. 2018 Jul 24;13(7):e0201178. doi: 10.1371/journal.pone.0201178 (PMC6057666; doi:10.1371/journal.pone.0201178)
Supplement: S1 Checklist — (DOCX) [file pone.0201178.s001.docx]

Consolidated criteria for reporting qualitative studies (COREQ): 32-item checklist

| **No** | **Item** | **Guide questions/description** |  | **Pg and line no.** |  |
| --- | --- | --- | --- | --- | --- |
| **Domain 1: Research team and reflexivity** | | | | | |
| Personal Characteristics | | | | | |
| 1. | Interviewer/facilitator | Which author/s conducted the interview or focus group? Ajay Patel |  |  |  |
| 2. | Credentials | What were the researcher's credentials? *E.g. PhD, MD*  PhD, LLB, Solicitor 10 years PQE |  |  |  |
| 3. | Occupation | What was their occupation at the time of the study? Senior lecturer |  |  |  |
| 4. | Gender | Was the researcher male or female? Male |  |  |  |
| 5. | Experience and training | What experience or training did the researcher have? Successful completion of research methods programme for PhD candidates, specialist training in qualitative methods and use of Nvivo |  |  |  |
| Relationship with participants | | | | | |
| 6. | Relationship established | Was a relationship established prior to study commencement? The participants were first contacted by email to request an interview |  |  |  |
| 7. | Participant knowledge of the interviewer | What did the participants know about the researcher? e*.g. personal goals, reasons for doing the research* Participants were informed that the study was being conducted in pursuance of a PhD study |  |  |  |
| 8. | Interviewer characteristics | What characteristics were reported about the interviewer/facilitator? e.g. *Bias, assumptions, reasons and interests in the research topic* The interviewer provided their reasons for the research but consciously avoided statements that might influence the participant’s views and in particular avoiding affirmation bias |  |  |  |
| **Domain 2: study design** | | | | | |
| Theoretical framework | | | | | |
| 9. | Methodological orientation and Theory | What methodological orientation was stated to underpin the study? *e.g. grounded theory, discourse analysis, ethnography, phenomenology, content analysis* Thematic analysis of phenomological study |  |  |  |
| Participant selection | | | | | |
| 10. | Sampling | How were participants selected? *e.g. purposive, convenience, consecutive, snowball* Purposive; elite interviews |  |  |  |
| 11. | Method of approach | How were participants approached? e*.g. face-to-face, telephone, mail, email* Email approach, actual interview face to face |  |  |  |
| 12. | Sample size | How many participants were in the study? |  |  |  |
| 13. | Non-participation | How many people refused to participate or dropped out? Reasons? 5. No reasons were given, there was no response. Reminders were sent but the researchers concluded that they were not prepared to participate and that it would be inappropriate to pursue them beyond this. |  |  |  |
| Setting | | | | | |
| 14. | Setting of data collection | Where was the data collected? e*.g. home, clinic, workplace* At the participant’s place of work |  |  |  |
| 15. | Presence of non-participants | Was anyone else present besides the participants and researchers? No |  |  |  |
| 16. | Description of sample | What are the important characteristics of the sample? *e.g. demographic data, date* Occupation, status and experience |  |  |  |
| Data collection | | | | | |
| 17. | Interview guide | Were questions, prompts, guides provided by the authors? Was it pilot tested? An interview guide was used. The first interview served as a pilot for those that followed |  |  |  |
| 18. | Repeat interviews | Were repeat interviews carried out? If yes, how many? No, they were not necessary |  |  |  |
| 19. | Audio/visual recording | Did the research use audio or visual recording to collect the data? Yes, a digital recorder was used and the recording was downloaded onto the secure drives of the institution |  |  |  |
| 20. | Field notes | Were field notes made during and/or after the interview or focus group? Contemporaneous notes were made during and after the interviews to provide corroboration and support for the audio recordings |  |  |  |
| 21. | Duration | What was the duration of the interviews or focus group? Between 45 – 90 minutes |  |  |  |
| 22. | Data saturation | Was data saturation discussed? Yes, refer line 67-9 where an explanation of the how data saturation was reached is provided |  |  |  |
| 23. | Transcripts returned | Were transcripts returned to participants for comment and/or correction? No. The interviews were clear, precise and uncontroversial and therefore the researchers did not need to return them for clarification. The interviewees were informed that the interview was to be transcribed and that they could check the transcript but there was no request to do so from them |  |  |  |
| **Domain 3: analysis and findings** | | | | | |
| Data analysis | | | | | |
| 24. | Number of data coders | How many data coders coded the data? One main coder but with some assistance and advice in checking from a more experienced researcher |  |  |  |
| 25. | Description of the coding tree | Did authors provide a description of the coding tree? The coding tree is in Nvivo and in the thesis |  |  |  |
| 26. | Derivation of themes | Were themes identified in advance or derived from the data? In advance |  |  |  |
| 27. | Software | What software, if applicable, was used to manage the data? Nvivo (10) |  |  |  |
| 28. | Participant checking | Did participants provide feedback on the findings? Participants were directed to an earlier publication based on the literature review <http://www.sciencedirect.com/science/article/pii/S0924224412001227?via%3Dihub> They were also invited to check their contributions (above) |  |  |  |
| Reporting | | | | | |
| 29. | Quotations presented | Were participant quotations presented to illustrate the themes / findings? Was each quotation identified? e*.g. participant number* Yes, each one was identified |  |  |  |
| 30. | Data and findings consistent | Was there consistency between the data presented and the findings? Yes, the findings were explicitly derived from the data |  |  |  |
| 31. | Clarity of major themes | Were major themes clearly presented in the findings? Yes, evidently. The major themes were clearly described and presented in the findings |  |  |  |
| 32. | Clarity of minor themes | Is there a description of diverse cases or discussion of minor themes? Yes, evidently. The minor themes were clearly described and presented in the findings |  |  |  |
